# Prevalence and Factors Associated with Acute Kidney Injury among Malaria Patients in Dar es Salaam: A Cross-Sectional Study

**DOI:** 10.1155/2019/4396108

**Published:** 2019-08-07

**Authors:** M. S. Muhamedhussein, S. Ghosh, K. Khanbhai, E. Maganga, Z. Nagri, M. Manji

**Affiliations:** ^1^Regency Medical Center, P.O. Box 2029, Dar es Salaam, Tanzania; ^2^Apollo Medical Centre, Dar es Salaam, Tanzania; ^3^Ebrahim Haji Charitable Health Centre, Dar es Salaam, Tanzania

## Abstract

**Introduction:**

Falciparum malaria still remains as a major cause of morbidity and mortality worldwide. Acute kidney injury is a known complication of malaria, and it is reported to occur in up to 40% of adult patients with severe falciparum malaria in endemic regions like sub-Saharan Africa.

**Objectives:**

To determine the prevalence and factors associated with acute kidney injury among falciparum malaria patients in a tertiary level private hospital in Dar es Salaam.

**Methodology:**

In a cross-sectional study design, 104 adults with falciparum malaria were enrolled consecutively over 6 months from April to September 2015. The diagnosis of acute kidney injury (AKI) in these patients was established using the KDIGO classification criteria. The prevalence of AKI was obtained at 48 hours from admission and at day 7. Different sociodemographic and clinical parameters which were associated with acute kidney injury at 48 hours and at day 7 were identified by hypothesis testing using chi squared tests followed by multivariate logistic regression analysis. Factors with a p value less than 0.05 were considered significant.

**Results:**

The participants were predominantly males 65.4% (68/104) and a third (36.5% (38/104)) were between 46 and 65 years. The prevalence of AKI among malaria patients at 48 hours was 26% (27/104). The prevalence of AKI among malaria patients at day 7 was 18.3% (19/104). On multivariate logistic regression, we found that factors that were significantly associated with AKI at 48 hours were male sex (OR 127, CI 3.4–4700, P = 0.008) and hemoglobin <7.5g/dl (OR 36.5, CI 1.7–797.7, P = 0.022), and factor associated with AKI at day 7 was baseline platelet count <25×10^3^ per mm^3^ (OR 77.8 CI 1.045–5798.6, P = 0.048). Only two patients needed hemodialysis (1.9%) and there were no deaths.

**Conclusion:**

Acute kidney injury is a common complication in patient with falciparum malaria. When managed well it has an excellent prognosis and necessitates dialysis in only a minority of patients. Male sex and hemoglobin is associated with AKI at 48 hours and baseline platelet count is associated with AKI at 7 days.

## 1. Introduction and Literature Review

Malaria which is caused by a Protozoan from genus Plasmodium is a global health problem with an estimated incidence of about 216 million worldwide causing 445,000 deaths in 2016 [1]. Four species of genus Plasmodium including falciparum, vivax, malaria, and ovale cause malaria when sporozoites enter the circulation and subsequently the liver after being bitten by a mosquito which is infected [2]. All species have similar clinical symptoms such as fever, chills, sweating, headache, nausea, and general weakness.

The most common laboratory abnormalities are thrombocytopenia, hyperbilirubinemia, and anemia [3]. Rapid malaria tests are now available and target histidine-rich protein 2 of P. falciparum or lactate dehydrogenase that is species specific. Their sensitivity is high but specificity is low [4]; therefore microscopy is commonly done to supplement the rapid tests [5].

Acute kidney injury (AKI) is one of the most dreaded complications of falciparum malaria. Resistance to drugs and increased virulence increases the chances of this and other complications of malaria [6–9].

Several hypotheses for pathogenesis of malaria associated kidney injury are proposed including mechanical obstruction by infected red blood cells and exaggerated immune response but exact mechanism is not known [10, 11].

Malaria associated acute kidney injury (MAKI) results in a wide variation of hospital admissions ranging from 2-39% and is predominantly seen in adults and is higher in areas which are nonendemic [12–14]. MAKI is documented to be more common in males than females [14].

In malaria endemic countries, up to 40% of patients with severe P. falciparum malaria can have acute kidney injury and mortality can be as high as 75% if renal replacement is delayed [15–18]. This kidney injury can be part of multiorgan dysfunction or can be the only organ injured and has better prognosis when renal injury is not accompanied with other organ failure [19].

In Cambodia, 23.3% of patients with malaria had acute kidney injury. A study done in India showed 35% of patients with severe malaria had malaria associated kidney injury [20]. Hypotension was found in 1/3 of patients diagnosed to have malaria [21]. One study in Ethiopia of patients with severe malaria showed a third of them had some degree of acute kidney injury while another showed 21% of consecutively acute kidney injured patients had Plasmodium falciparum infection [22, 23]. Anemia, thrombocytopenia, and proteinuria also frequently accompany AKI in such patients [13, 14]. Mortality of malaria associated kidney injury is reported to be as high as 39.7%.

Artemisinin based combination therapy (ACT) is the mainstay of antimalarial treatment as advocated by the WHO. The Tanzania national guidelines for the diagnosis and treatment of malaria also advocate similarly [24, 25].

There is little or no data regarding MAKI in the country although it remains a common clinical scenario in everyday practice. With this study we hope to obtain an idea of its prevalence and any significant clinical and demographic factors that may coexist around MAKI.

## 2. Methodology

### 2.1. Study Design and Setting

A cross-sectional study was conducted for a period of six months from April 2015 to September 2015 at Regency Medical Center, Dar es Salaam. Regency medical centre is one of the tertiary private hospitals in Dar es Salaam. It has an established nephrology and internal medicine department that attend to the patients enrolled in the study. Other auxiliary departments that were also involved in the care of such patients are the intensive care unit and the dialysis unit. Laboratory services were provided by Lancet Laboratories which is SANAS (South African National Accreditation System) accredited.

### 2.2. Data Collection

All patients above 18 years with clinical features of malaria presenting at outpatient and inpatient basis during the study duration were tested for malaria using the ParaHit rapid test. Once malaria antigen was found to be positive, thick and thin smear were prepared. Thin smear was done by spreading the blood on the specimen slide using the spreader slide at 30-45 degree, dried, and fixed with methyl alcohol. Thick smear was prepared by Field's stain A and B. A drop of blood was put on a slide and air dried then dipped in Field stain A for 3 seconds then dipped into tap water for a further 3 seconds. After that the slide is dipped in Field stain B for 3 seconds and tapped with water to remove excess stain. The slide was then left to dry and then examined under a microscope. Malaria percentage was calculated using the number of parasites in 10 fields of 100 Red blood cells. For example, if the total number of parasites were 25 in 10 fields of 100 Red blood cells, then malaria parasitemia was 2.5%.

Renal functions were assessed for each of these patients by checking serum creatinine and urea. The KDIGO classification criteria were used to diagnose acute kidney injury [26]. Urinalysis was done using both dipsticks and microscopy. For all patients follow-up renal function tests were done on day 2 and day 7 and after 3 months.

All patients received the standard protocol directed therapy for malaria which included an artemisinin based therapy as per the Tanzanian national guidelines [24] for management of malaria. Patients diagnosed with acute kidney injury were managed by a nephrologist according to best medical practice established for AKI patients. This included avoidance of nephrotoxic drugs, renal dosing of antibiotics when needed, correction of electrolyte, and acid base abnormalities and inotropes when clinically indicated for hypotension. The need for fluid replacement versus diuresis was assessed as per the clinical scenario of the patient and this was decided by the nephrologist, intensivist, or physician at the time. This was based on clinical signs of hydration like skin turgor and capillary refill time, urine output, blood pressure, and other vital parameters, evidence of pulmonary congestion clinically or based on chest X-ray, or jugular venous characteristics as assessed clinically. Hypovolemic patients were given the standard fluid challenge of 20ml/kg of 0.9% normal saline over the first 60 minutes and lungs were checked for overload. After fluid replacement furosemide at 40 mg was given to challenge the kidneys initially. This was then given at intervals if no urine output was noted. Patients were initiated on hemodialysis when complications of AKI were not correctable medically. These included uremia, refractory fluid overload, and refractory hyperkalemia. Jugular or femoral veins were used for venous access for dialysis.

### 2.3. Definition of Terms

We have used the KDIGO criteria which define acute kidney injury as increase in serum creatinine by ≥0.3md/dl (26.4 mol/L) in 48 hours or increase in serum creatinine ≥ 1.5X from baseline within 7 days [26].

### 2.4. Statistical Analysis

The prevalence of AKI was assessed at 48 hours and at day 7. Factors associated with AKI at 48 hours and day 7 were identified using chi square tests, followed by logistic regression models for factors that were significant. Variables that had a p value < 0.05 on multivariate analysis were considered significant for AKI among malaria patients.

### 2.5. Ethical Considerations

Permission to conduct the study was obtained from the hospital, the regional and district authorities, and consent was also taken from the patients.

## 3. Results

The participants were predominantly males 65.4% (68/104) and a third (36.5% (38/104)) were between 46 and 65 years. Majority of the patient had no comorbid conditions (73.1% (76/104)) and the most commonly sighted comorbidity was hypertension (10.56% (11/104)) and diabetes (5.8% (6/104)) (Table 1).

At admission almost half (47.1% (49/104)) had malaria parasitemia between 1.1 and 5% while 5.8% (6/104) and 8.7% (9/104) had malaria parasitemia of 5.1%-10% and >10%, respectively (Table 1).

Twenty-four patients (23.1% (24/104)) were anemic with hemoglobin of less than 11g/dL on admission, 6 of whom had hemoglobin levels less than 7.5g/dL. Ninety out of 104 patients (86.6%) had thrombocytopenia, out of which 8 patients had platelets less than 25 X 10^9^/L (Table 1).

At admission the mean creatinine ± SD was 144 ± 95.6 mmol/L. Control creatinine values at day 2, day 7, and day 90 were 140 ± 169.4, 98 ± 87.5, and 80 ± 37 mmol/L, respectively. Proteinuria was found in 69.2% of patients out of which 3.8% had protein of 3+ of urine dipstick. Hematuria was found in 38.4% from which 6.7% had blood of 3+ by dipstick (Table 1).

Eight patients (7.6%) received transfusion of blood and blood products of which 3 patients received whole blood, 4 patients received platelets, and 1 patient received both. All patients were treated with artemisinin based combination therapy as per the current national guidelines for malaria. Only 2 patients (1.9%) had to undergo dialysis for the AKI. There were no deaths in the study (Table 1).

The prevalence of AKI among malaria patients at 48 hours was 26% (27/104). The prevalence of AKI among malaria patients at day 7 was 18.3% (19/104). Out of 27 patients who had AKI at 48hrs, 8 patients had recovered by day 7 (29.6%). Out of 27 patients who had AKI at 48hrs, 14 patients had recovered by 3 months (51.8%) (Table 2).

Individual associations between AKI and sex, parasitemia, hemoglobin, platelet count, and urine protein were significant (Table 3). These were further analyzed in logistic regression. On multivariate logistic regression, we found that factors that were significantly associated with AKI at 48 hours were male sex (OR 127, CI 3.4–4700, P = 0.008) and hemoglobin <7.5g/dl (OR 36.5, CI 1.7–797.7, P = 0.022) and factors that were significantly associated with AKI at 7 days were baseline platelet count <25×10^3^ per mm^3^ (OR 77.8 CI 1.045–5798.6, P = 0.048) (Table 4).

## 4. Discussion

This study has found that almost 26% of patients admitted with malaria have AKI within the first 48 hours. This dropped to 18.3% at day 7. The most important factor associated with AKI at 48 hours was male sex and hemoglobin < 7.5g/dl and the most important factor associated with AKI at day 7 was baseline platelet count <25X10^3^/mm^3^.

The prevalence of AKI from malaria in this study is in keeping with the prevalence reported in other settings (2-39%) [12–14] albeit at a higher end of this spectrum. Given the endemic nature of malaria in this setting, this prevalence is expected to prevail if not increase in the foreseeable future. Despite this high prevalence, it has an excellent prognosis when managed well.

The parasite burden at admission was significantly associated with AKI at 48hrs and at day 7. This is not surprising given the fact that the mechanisms responsible for AKI in malaria patients include immunological insults to the kidney that are derived from host-parasite immunological reactions [10, 11]. A higher parasite burden would provoke a more intense immunological reaction in the host and thus higher likelihood of AKI. Despite this, it is also important to note the high potency of artemisinin based combination in achieving parasite clearance. One study has reported a parasite clearance time of 30 hours when artesunate is used [27]. With such rapid clearance, it is possible that while parasite burden may seem important in AKI early on in the infection, it loses its strength over other perhaps more potent factors like anemia and thrombocytopenia. It is thus not surprising that it was not significant in our eventual regression analysis.

Anemia and thrombocytopenia were also important factors associated with AKI both at 48 hours and day 7. Both anemia and thrombocytopenia are standalone complications of severe malaria and as such highlight the coexistent severity at presentation of such patients. Anemia contributes to circulatory insufficiency and hypoxic state that has the potential to worsen the renal impairment in MAKI. Severe thrombocytopenia may predispose to systemic bleeding leading to hypotension or shock or renal hemorrhage that will worsen the AKI. Improvement in platelet counts upon institution of appropriate treatment is frequently used a clinical parameter to gauge treatment response to the systemic symptoms of malaria by clinicians. As per our logistic regression analysis, a hemoglobin less than 7.5g/dl was a strong predictor of AKI at 48 hours while baseline platelet count held its value up to day 7 in predicting AKI.

Prior coexistent comorbidities like hypertension, diabetes, and HIV were not associated with AKI either at 48 hours or at day 7. Urine for protein is another important noninvasive proxy of the changes that happen to the kidney during malaria. In this study we found that proteinuria at admission was significantly associated with AKI at 48 hours and at day 7. However it lost its significance when analyzed in regression models along with other clinical characteristics.

CRP as a marker of inflammation was not found to be associated with AKI at 48 hours or at day 7. The mechanisms of nephroinflammation in MAKI are different compared to pathologic mechanisms of systemic inflammation in AKI due to bacterial sepsis where determination of acute phase proteins may hold more value.

Among the common clinical dilemmas in MAKI is when to institute dialysis. In this study, our approach was to dialyse patients based on clinical indications rather than just basing the decision on the patient's creatinine. So while changes in the patient's creatinine were used to diagnose AKI, creatinine alone was not used as criteria for starting dialysis. In this entire sample, only 2 (1.9%) patients had to be started on dialysis. In both instances, the reason for initiation was uremic encephalopathy and altered mental status. This rapidly corrected after dialysis and both patients did not require more than 3 sessions.

There was no reported mortality from AKI in this study. This study was done in a private tertiary level hospital in a major city in Tanzania. The facility has excellent diagnostic and super specialty level nephrology and intensive care. The situation may not be the same in most parts of Tanzania where such facilities are not available. We therefore think that the prevalence of MAKI may actually be higher elsewhere in Tanzania, and the zero mortality shown from this study may actually be an under representation of the true scenario in the country. If anything, we think that this study sheds light on the problem of MAKI that has not received the attention it requires at a national level.

This study was mainly limited by its small sample size. Analysis of markers of recovery at day 7 and day 30 were not performed due to small numbers. Kidney biopsies were not done to assess histological characteristics of MAKI. This was beyond the scope of this study. The diagnostic criteria for the diagnosis of MAKI depend on dynamic changes in creatinine levels over time. As such a diagnosis of the level of AKI at admission was impossible. It is possible that a few patients who had renal insults at admission may have recovered by 48 hours and who have gone undetected on this study.

## 5. Conclusion

Acute kidney injury is a common complication in patient with falciparum malaria. When managed well it has an excellent prognosis and necessitates dialysis in only a minority of patients. Male sex and hemoglobin < 7.5g/dl are associated with AKI at 48 hours and baseline platelet count <25X10^3^/mm^3^ is associated with AKI at 7 days.

## Figures and Tables

**Table 1:** Sociodemographic and clinical characteristics.

Characteristics	Frequency	Percent	Mean ± SD
Age			
18 -25	31	29.8	38.65±16.1
26 -45	27	26.0	
46 -65	38	36.5	
>65	8	7.7	
Sex			
Male	68	65.4	
Female	36	34.6	
Comorbidities			
None	76	73.1	
Hypertension	11	10.6	
Diabetes	6	5.8	
HIV	2	1.9	
Others	9	8.7	
Parasitemia at admission			
<0.5%	12	11.5	
0.5-1.0%	28	26.9	
1.1-5%	49	47.1	
5.1-10%	6	5.8	
>10%	9	8.7	
Creatinine at admission			144 ± 95.6
Creatinine at day 2			140 ± 169.4
Creatinine at day 7			98 ± 87.5
Creatinine at day 90			80 ± 37
Hemoglobin at admission (g/dl)			
<7.5	6	5.8	12.12±2.21
7.7-9	4	3.8	
9.1-11	14	13.5	
>11	80	76.9	
Platelet at admission			
<25	8	7.7	96.9±69
25-50	19	18.3	
51-100	39	37.5	
101-150	24	23.1	
>150	14	13.5	
Urine for protein			
None	17	16.3	
Trace	20	19.2	
1 – 2+	58	55.8	
>3+	9	8.7	

**Table 2:** The prevalence of AKI.

AKI	Yes	No	Total
At 48 hours	27 (26%)	77 (74%)	104 (100%)
At 7 days	19 (18.3%)	85 (81.7%)	104 (100%)

**Table 3:** Factors associated with AKI in malaria patients at 48 hours and at day 7.

Parameter	Variable	AKI at 48h	P value	AKI at 7days	P value
Age group (yrs)	18 – 25	8 (29.8%)	0.883	5 (26.3%)	0.514
	26 – 45	7 (25.9%)		4 (21.1%	
	46 – 65	9 (33.3%)		7 (36.8%)	
	> 65	3 (11.1%)		3 (15.8%)	

Sex	Male	24 (88.9%)	0.003	18 (94.7%)	0.003
	Female	3 (11.1)		1 (5.3%)	

Comorbids	None	18 (66.7%)	0.745	12 (63.2%)	0.669
	Hypertension	4 (14.8%)		3 (15.8%)	
	Diabetes	1 (3.7%)		1 (5.3%)	
	Others	3 (11.1%)		1 (5.3%)	

Parasitemia (%)	<0.5%	1 (3.7%)	0.004	1 (5.3%)	0.046
	0.5-1.0%	7 (25.9%)		5 (26.3%)	
	1.1-5%	11 (40.7%)		7 (36.8%)	
	5.1-10%	1 (3.7%)		1 (5.3%)	
	>10%	7 (25.9%)		5 (26.3%)	

Hemoglobin (g/dl)	<7.5	4 (14.8%)	0.010	2 (10.5%)	0.017
	7.7-9	3 (11.1%)		3 (15.8%)	
	9.1-11	3 (11.1%)		2 (10.5%)	
	>11	17 (63.0%)		12 (63.2%)	

Platelets *∗*10^3^/mm^3^	<25	7 (25.9%)	<0.001	7 (36.8%)	<0.001
	25-50	6 (22.2%)		4 (21.1%)	
	51-100	10 (37.0%)		4 (21.1%)	
	101-150	2 (7.4%)		2 (10.5%)	
	>150	2 (7.4%)		2 (10.5%)	

Urine protein	None	3 (11.1%)	0.001	2 (10.5%)	<0.001
	Trace	2 (7.4%)		2 (10.5%)	
	1 – 2 +	15 (55.6%)		8 (42.1%)	
	3 +	7 (25.9%)		7 (36.8%)	

CRP (mg/L)	<4	1 (3.7%)	0.126	1 (5.3%)	0.223
	4-10	2 (7.4%)		1 (5.3%)	
	11-50	3 (11.1%)		2 (10.5%)	
	>50	21 (77.8%)		15 (78.9%)	

**Table 4:** Logistic regression analysis of factors associated with AKI at 48 hours and day 7.

			AKI at 48 hours			AKI at 7 days		
Parameter	Variable	Comparison	Adj OR	CI	P value	Adj OR	CI	P value
Sex	Male	Female	127.7	3.4 – 4700.8	0.008	56.5	0.76 - 4201	.066

Parasitemia (%)	0.5-1.0%	<0.5%	2.566	.158 – 41.8	0.508	3.534	0.16 – 79.5	0.427
	1.1-5%		2.334	.153 – 35.6	0.542	.212	0.004 -10.6	0.437
	5.1-10%		4.798	.132 – 174.5	0.392	17.567	.252 – 1223.6	0.186
	>10%		119.4	.430 - 33148	0.096	2.610	0.036 – 187.6	0.660

Hemoglobin (g/dl)	<7.5	>11	36.5	1.7 – 797.7	0.022	20.790	0.8 – 493.8	0.060
	7.7-9		56.4	.5 – 6924.8	0.1	1065.345	0.53 – 2143427.3	0.072
	9.1-11		28.2	.7 – 1149.8	0.078	18.539	0.29 – 1167.1	0.167

Platelets *∗*10^3^/mm^3^	<25	>150	19.2	.6 – 528.5	0.081	77.826	1.04 – 5798.6	0.048
	25-50		2.9	.2 – 41.7	0.421	0.737	0.03 – 19.6	0.855
	51-100		.718	0.05 – 8.9	0.797	0.081	0.002 – 3.2	0.181
	101-150		.093	0.003 – 2.52	0.158	0.122	0.003 – 4.8	0.262

Urine protein	None	Trace	0.334	0.029 – 3.8	0.380	0.467	0.03 – 8.8	0.611
	1 – 2+		1.127	.159 – 8.0	0.905	0.488	0.03 – 7.5	0.606
	3+		1.084	0.059 -20.0	0.957	5.857	0.14 – 254.8	0.359

## Data Availability

The data used to support the findings of this study are available from the corresponding author upon request.
